# Impact of baseline clinical asthma characteristics on the response to mepolizumab: a post hoc meta-analysis of two Phase III trials

**DOI:** 10.1186/s12931-021-01767-z

**Published:** 2021-06-22

**Authors:** Catherine Lemiere, Camille Taillé, Jason Kihyuk Lee, Steven G. Smith, Stephen Mallett, Frank C. Albers, Eric S. Bradford, Steven W. Yancey, Mark C. Liu

**Affiliations:** 1 Faculty of Medicine, Université de Montréal, Montreal, QC Canada; 2 Hôpital du Sacré-Cœur de Montréal, Montreal, QC Canada; 3 Service de Pneumologie, Hôpital Bichat, AP-HP-Nord, Paris, France; 4 INSERM U1152, Université de Paris, Paris, France; 5 INSERM 12, F-CRIN, Clinical Research Initiative In Severe Asthma: A Level for Innovation & Science (CRISALIS), Toulouse, France; 6 Toronto Allergy and Asthma Clinic, Toronto, ON Canada; 7 Respiratory Therapeutic Area, GlaxoSmithKline, Research Triangle Park, NC USA; 8 Clinical Statistics, GlaxoSmithKline, Stockley Park, Uxbridge, UK; 9 Respiratory Medical Franchise, GSK, Research Triangle Park, NC USA; 10 Divisions of Allergy and Clinical Immunology, Pulmonary and Critical Care Medicine, Johns Hopkins Asthma and Allergy Center, 5501 Hopkins Bayview Circle, Baltimore, MD 21224 USA; 11 Present Address: Avillion US Inc., Northbrook, IL USA; 12 Present Address: Aeglea BioTherapeutics, Austin, TX USA

**Keywords:** Asthma, Mepolizumab, Asthma control, Allergen sensitivity, Lung function, Airway reversibility, Exacerbations, Health-related quality of life

## Abstract

**Background:**

Severe asthma is associated with a broad range of phenotypes and clinical characteristics. This analysis assessed whether select baseline patient characteristics could prognosticate mepolizumab efficacy in severe eosinophilic asthma.

**Methods:**

This was a post hoc meta-analysis of data from the Phase III MENSA (NCT01691521/MEA115588) and MUSCA (NCT02281318/200862) studies. Patients aged ≥ 12 years with severe eosinophilic asthma and a history of exacerbations were randomised to receive placebo (MENSA/MUSCA), mepolizumab 75 mg intravenously (MENSA) or 100 mg subcutaneously (SC) (MENSA/MUSCA) every 4 weeks for 32 (MENSA) or 24 (MUSCA) weeks. The primary endpoint was the annual rate of clinically significant exacerbations; other outcomes included the proportion of patients with no exacerbations and changes from baseline in pre-bronchodilator forced expiratory volume in 1 s (FEV_1_), St George’s Respiratory Questionnaire (SGRQ) total score and Asthma Control Questionnaire (ACQ)-5 score. Analyses were performed by baseline age of asthma onset (< 18 years; 18–40 years; ≥ 40 years); lung function (% predicted FEV_1_ ≤ 60; 60–80; > 80); airway reversibility (reversible [≥ 12% change in FEV_1_]; non-reversible [< 12% change in FEV_1_]); perennial and/or seasonal allergen sensitivity (yes/no); asthma control (uncontrolled [ACQ-5 score ≥ 1.5]; partial/complete control [ACQ-5 score < 1.5]).

**Results:**

Overall, 936 patients received mepolizumab 100 mg SC or placebo. Across age at asthma onset, lung function and airway reversibility subgroups, mepolizumab reduced the rate of clinically significant exacerbations by 49–63% versus placebo. Improvements in lung function, SGRQ total score and ACQ-5 score were also seen with mepolizumab versus placebo across most age and lung function subgroups. Clinically significant exacerbations were reduced with mepolizumab versus placebo irrespective of season or allergen sensitivity; SGRQ total and ACQ-5 scores were generally improved across seasons.

**Conclusions:**

Mepolizumab efficacy was consistent for patients with varying age at asthma onset, lung function, airway reversibility and allergen sensitivities at baseline. Our results indicate that mepolizumab is likely to be beneficial for patients with severe eosinophilic asthma with a broad range of baseline clinical characteristics; large-scale real-world studies are needed to confirm the external validity of these findings.

*Trial registration* Post hoc meta-analysis of data from MENSA (NCT01691521/MEA115588) and MUSCA (NCT02281318/200862)

## Background

Severe asthma affects around 3–10% of the asthma population, and is characterised by frequent, persistent respiratory symptoms, in spite of regular use of maintenance therapies and additional controllers [1, 2]. Patients with severe eosinophilic asthma, a phenotype of severe asthma, have persistent eosinophilic airway inflammation and experience recurrent exacerbations [1, 3].

Mepolizumab is a targeted, humanised anti-interleukin (IL)-5 monoclonal antibody that prevents IL-5 from binding to its receptor mainly on eosinophils, and selectively inhibits eosinophilic inflammation [4]. It is currently approved for the treatment of severe eosinophilic asthma in patients ≥ 6 years of age in multiple regions worldwide and for the treatment of eosinophilic granulomatosis with polyangiitis in adults in the USA [5, 6]. During its Phase III clinical development programme, mepolizumab was shown to reduce exacerbation rates, decrease oral glucocorticoid dependence, and improve lung function, health-related quality of life (HRQoL) and asthma control, versus placebo, in patients with severe eosinophilic asthma [7–10].

Defining severe asthma subtypes using clinical and/or laboratory-based biomarkers can help to inform treatment decisions in clinical practice. For example, data from clinical studies and post hoc analyses suggest that baseline blood eosinophil counts are predictive of response to mepolizumab treatment [10–13]. In addition, a recent history of exacerbations and uncontrolled asthma are useful characteristics in the assessment and prognostication of treatment outcomes in patients with severe eosinophilic asthma [14]. Owing to the heterogenous nature of severe asthma, it is of clinical interest to investigate whether any other patient characteristics can affect the response to mepolizumab treatment; knowledge of these characteristics could assist clinicians involved in patient management.

The aim of this post hoc meta-analysis of data from two Phase III trials, MENSA (MEA115588/NCT01691521) [9] and MUSCA (200862/NCT02281318) [8] was to assess the relationship between the response to mepolizumab treatment and various baseline clinical characteristics, such as asthma control, age of asthma onset, allergen sensitivity, and lung function.

## Methods

### Study design

This was a post hoc meta-analysis of data from two Phase III, placebo-controlled, randomised, double-blind, parallel-group, multicentre studies, MENSA and MUSCA [8, 9]. Full details of these studies have been published previously [8, 9]. Briefly, during MENSA, patients were randomised (1:1:1) to receive mepolizumab 75 mg intravenously (IV), mepolizumab 100 mg administered subcutaneously (SC) or placebo, plus optimised standard of care (high-dose inhaled corticosteroids [ICS] and another controller), every 4 weeks for 32 weeks. Patients enrolled in MUSCA were randomised (1:1) to receive mepolizumab 100 mg SC or placebo, plus standard of care, every 4 weeks for 24 weeks. The protocols for MENSA and MUSCA are available online from the GSK Clinical Study Register [15]. This post hoc analysis reports data from patients who received placebo or mepolizumab 100 mg SC only.

MENSA and MUSCA were conducted in accordance with the ethical principles of the Declaration of Helsinki, International Conference on Harmonisation Good Clinical Practice Guidelines, and applicable country-specific regulatory requirements. All patients provided written informed consent.

### Patients

Patients enrolled in MENSA and MUSCA were ≥ 12 years of age with severe eosinophilic asthma (blood eosinophil count ≥ 300 cells/µL in the previous year, or ≥ 150 cells/µL at screening) and a history of ≥ 2 exacerbations requiring systemic corticosteroids in the year prior to enrolment despite receiving optimised standard of care therapy (regular treatment with high-dose ICS in the 12 months prior to screening, plus ≥ 1 additional controller medication with or without oral corticosteroids [OCS] for ≥ 3 months). Patients < 18 years of age were required to have a forced expiratory volume in 1 s (FEV_1_) < 90% predicted or a FEV_1_/forced vital capacity (FVC) ratio < 0.8, whilst those ≥ 18 years of age were required to have a FEV_1_ < 80% predicted. This was in addition to one or more of the following: FEV_1_ reversibility ≥ 12%, positive methacholine or mannitol challenge results at visit 1 or 2 or during the prior year, and FEV_1_ variability (≥ 20%) between two clinic visits in the past 12 months.

### Endpoints and assessments

The primary endpoint was the annual rate of clinically significant exacerbations, defined as a worsening of asthma requiring the use of systemic corticosteroids and/or hospitalisation/emergency department (ED) visit. Secondary endpoints included: the proportion of patients with no clinically significant exacerbations over the course of the study; change from baseline in pre-bronchodilator FEV_1_ at study end; change from baseline in St George’s Respiratory Questionnaire (SGRQ) total score at study end; change from baseline in Asthma Control Questionnaire (ACQ)-5 score, during 4-week periods and at study end; the proportion of patients achieving a ≥ 4-point improvement from baseline in SGRQ total score at study end; the proportion of patients achieving a ≥ 0.5-point improvement from baseline in ACQ-5 score at study end; the proportion of patients achieving complete asthma control (ACQ-5 score < 0.75) at study end; the proportion of patients per month with a clinically significant exacerbation.

### Sample size and statistical analysis

This meta-analysis included data from patients who received ≥ 1 dose of either placebo or mepolizumab (100 mg SC); the modified intent-to-treat population. Analyses were stratified by disease characteristics at baseline: age of asthma onset (< 18 years; 18–40 years; ≥ 40 years); lung function (% predicted FEV_1_ ≤ 60; 60–80; > 80); airway reversibility (reversible [≥ 12% change in FEV_1_]; non-reversible [< 12% change in FEV_1_]); allergen sensitivity (sensitivity to ≥ 1 perennial allergen [yes/no]; sensitivity to ≥ 1 seasonal allergen [yes/no]); asthma control (uncontrolled [ACQ-5 score ≥ 1.5]; partial/complete control [ACQ-5 score < 1.5]). For the allergen sensitivity subgroups, blood tests for immunoglobulin (Ig)E against specific perennial and seasonal allergens were performed. The perennial allergens tested were *Dermatophagoides farinae, Dermatophagoides pteronyssinus*, dog epithelium, cat epithelium and *Alternaria tenuis;* the seasonal allergens tested were elm, olive tree, oak white, thistle, wild rye, Bermuda grass and western ragweed pollen. Any IgE responses to these allergens ≥ 0.35 kU/L were considered positive. Seasons were classified as spring (March/April/May), summer (June/July/August), autumn (September/October/November) and winter (December/January/February) for patients enrolled in the northern hemisphere. For patients enrolled in the southern hemisphere, 6 months were added to the exacerbation start date to classify the season.

The rate of exacerbations was analysed separately for each subgroup in each study using a negative binomial model. Continuous endpoints were analysed using a mixed model repeated measures analysis. The proportion of patients with no clinically significant exacerbations was analysed using a logistic regression model. In all model-based analyses, terms for treatment group, geographical region, baseline maintenance OCS use (yes/no), baseline % predicted pre-bronchodilator FEV_1_, and exacerbations in the year prior to the study (2, 3, 4+; as an ordinal variable) were included as covariates. When a baseline value for the analysis variable was available, this was also included as a covariate for analyses with continuous endpoints. Baseline % predicted FEV_1_ was not included in the analyses of pre/post-bronchodilator FEV_1_; instead the relevant absolute baseline value was used. The study visit, plus interaction terms for visit by baseline and visit by treatment group were included as covariates for analyses of endpoints with repeated measures. End-of-study treatment differences between mepolizumab 100 mg SC and placebo for each subgroup were combined across studies using an inverse variance weighted fixed-effects meta-analysis. The protocol for this meta-analysis is available on the GSK Clinical Studies Register (Study ID 208115) [15].

## Results

### Patient population

Of the 936 patients included in this meta-analysis, 468 received placebo and 468 received mepolizumab 100 mg SC. Patient demographics and characteristics for the overall population, as well as the proportions of patients receiving mepolizumab or placebo in each baseline characteristic subgroup, are shown in Table 1. In general, baseline characteristics subgroups were well matched by age; as expected based on the trials’ inclusion criteria, patients in the > 80% predicted FEV_1_ subgroup were younger compared with other subgroups (data not shown).

**Table 1:** Summary of baseline demographics, clinical characteristics, and analysis subgroups

	Mepolizumab (100 mg SC)	Placebo	Total
	N = 468	N = 468	N = 936
Overall population			
Mean (SD) age, years	50.4 (14.25)	50.9 (13.55)	50.6 (13.90)
Female, n (%)	265 (57)	283 (60)	548 (59)
Mean (SD) % predicted FEV_1_	58.9 (16.74)	60.1 (16.93)	59.5 (16.84)
Mean (SD) SGRQ total score	47.6 (18.64)	46.5 (19.25)	47.1 (18.94)
Mean (SD) ACQ-5 score	2.25 (1.17)	2.21 (1.18)	2.23 (1.17)
Receiving maintenance OCS therapy, n (%)	116 (25)	111 (24)	227 (24)
Exacerbations in previous year, n (%)			
2	248 (53)	273 (58)	521 (56)
3	96 (21)	94 (20)	190 (20)
≥ 4	124 (26)	101 (22)	225 (24)
Geometric mean (SD log) eosinophil count, cells/μL	320 (0.965)	340 (0.929)	330 (0.947)
Subgroups, n (%)			
Age of asthma onset ( years)			
< 18	132 (28)	122 (26)	254 (27)
18– < 40	173 (37)	172 (37)	345 (37)
≥ 40	162 (35)	174 (37)	336 (36)
Lung function			
≤ 60% predicted FEV_1_	245 (52)	244 (52)	489 (52)
> 60–80% predicted FEV_1_	177 (38)	177 (38)	354 (38)
> 80% predicted FEV_1_	46 (10)	47 (10)	93 (10)
Airway reversibility			
n^a^	457	460	917
Reversible^b^	312 (68)	299 (65)	611 (67)
Non-reversible^c^	145 (32)	161 (35)	306 (33)
Allergen sensitivity			
n^a^	454	457	911
Sensitivity to ≥ 1 allergen	215 (47)	216 (47)	496 (54)
Sensitivity to ≥ 1 seasonal allergen	144 (32)	146 (32)	290 (32)
Asthma control			
n^a^	465	462	927
Uncontrolled asthma^d^	334 (72)	314 (68)	648 (70)
Partially or fully controlled asthma^e^	131 (28)	148 (32)	279 (30)

*ACQ-5* Asthma Control Questionnaire, 5 item, *FEV*_*1*_ forced expiratory volume in 1 s, *SC* subcutaneous, *SD* standard deviation

^a^Not all patients had data available on airway reversibility, asthma control and allergen sensitivity; those without ≥ 1 result were not included in the relevant subanalyses

^b^Defined as patients with a ≥ 12% change in FEV_1_

^c^Defined as patients with a < 12% change in FEV_1_

^d^Defined as an ACQ-5 score ≥ 1.5

^e^defined as an ACQ-5 score < 1.5

### Age of asthma onset subgroups

**Fig. 1:**
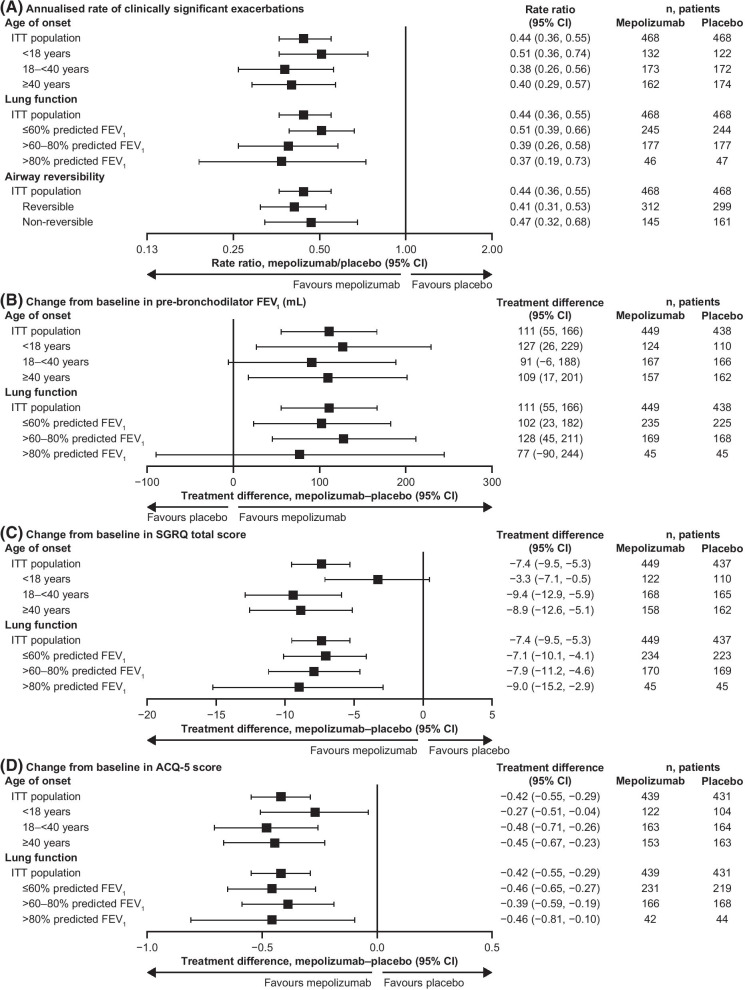
Efficacy of mepolizumab, by age at asthma onset, lung function, and airway reversibility. **A** Annualised rates of clinically significant exacerbations; **B** Change from baseline to study end in pre-bronchodilator FEV_1_; **C** Change from baseline to study end in SGRQ total score; **D** Change from baseline to study end in ACQ-5 score. All plots are for patients receiving mepolizumab versus placebo. *ACQ* Asthma Control Questionnaire, *CI* confidence interval, *FEV*_*1*_ forced expiratory volume in 1 sm *ITT* intent-to-treat, *SGRQ* St George’s Respiratory Questionnaire

Across all age of asthma onset subgroups, mepolizumab was associated with reductions of 49–62% in the annual rate of clinically significant exacerbations compared with placebo (Fig. 1A). Reductions in the annual rate of exacerbations requiring ED visits/hospitalisations were also seen with mepolizumab versus placebo across all age of asthma onset subgroups (Table 2). Patients receiving mepolizumab were more likely to experience no clinically significant exacerbations during the study than those who received placebo, regardless of age of asthma onset (Table 2). Mepolizumab also resulted in an increase from baseline in pre-bronchodilator FEV_1_ versus placebo in all age of asthma onset subgroups (Fig. 1B). Improvements from baseline in SGRQ total score with mepolizumab versus placebo were seen in all age of asthma onset subgroups, the greatest differences observed in the 18–< 40 years (treatment difference [95% confidence interval] − 9.4 [− 12.9, − 5.9]) and ≥ 40 years (treatment difference [95% CI] − 8.9 [− 12.6, − 5.1]) subgroups (Fig. 1C). Furthermore, the proportion of patients achieving the minimal clinically important difference (MCID) of a 4-point improvement from baseline in SGRQ total score was higher with mepolizumab versus placebo, regardless of age at asthma onset (Table 3). Improvements in ACQ-5 score with mepolizumab versus placebo were also seen regardless of age at asthma onset (Fig. 1D), and a higher proportion of patients receiving mepolizumab achieved the MCID of a 0.5-point improvement from baseline in ACQ-5 score, compared with those receiving placebo, across all age at asthma onset subgroups (Table 4).

**Table 2:** Clinically significant exacerbations, by baseline characteristic subgroup

	Odds ratio, mepolizumab/placebo (95% CI)	Number of patients	
		Mepolizumab	Placebo
Annualised rate of exacerbations requiring an ED visit/hospitalisation			
Age at asthma onset			
ITT population	0.36 (0.20, 0.67)	468	468
< 18 years	0.23 (0.08, 0.69)	132	122
18–< 40 years	0.21 (0.05, 0.83)	173	172
≥ 40 years	0.51 (0.19, 1.35)	162	174
Lung function			
ITT population	0.36 (0.20, 0.67)	468	468
≤ 60% predicted FEV_1_	0.41 (0.19, 0.86)	245	244
> 60–80% predicted FEV_1_	Non-est*	n/a	n/a
≥ 80% predicted FEV_1_	Non-est*	n/a	n/a
% patients with no clinically significant exacerbations			
Age at asthma onset			
ITT population	3.03 (2.25, 4.07)	468	468
< 18 years	2.97 (1.67, 5.26)	132	122
18–< 40 years	3.19 (1.90, 5.36)	173	172
≥ 40 years	3.32 (1.98, 5.55)	162	174
Lung function			
ITT population	3.03 (2.25, 4.07)	468	468
≤ 60% predicted FEV_1_	2.67 (1.79, 3.98)	245	244
> 60–80% predicted FEV_1_	3.18 (1.92, 5.28)	177	177
≥ 80% predicted FEV_1_	4.90 (1.54, 15.62)	46	47

*CI* confidence interval, *ED* emergency department, *FEV*_*1*_ forced expiratory volume in 1 s, *ITT* intent-to-treat, *n/a* not applicable, *non-est* non-estimable

^a^The odds ratio was non-estimable for this subgroup owing to insufficient data to perform modelling

**Table 3:** Proportion of patients achieving ≥ 4-point reductions in SGRQ total score, by baseline characteristics

	Mepolizumab		Placebo
ITT population			
Patients with ≥ 4-point reduction in SGRQ total score, n (%)	335/466 (72)		256/465 (55)
Odds ratio (mepolizumab/placebo), 95% CI; p-value		2.17 (1.63, 2.87); p < 0.001	
Age at asthma onset: < 18 years			
Patients with ≥ 4-point reduction in SGRQ total score, n (%)	90/130 (69)		65/122 (53)
Odds ratio (mepolizumab/placebo), 95% CI		1.95 (1.12, 3.38)	
Age at asthma onset: 18–< 40 years			
Patients with ≥ 4-point reduction in SGRQ total score, n (%)	123/173 (71)		96/171 (56)
Odds ratio (mepolizumab/placebo), 95% CI		2.17 (1.34, 3.51)	
Age at asthma onset: ≥ 40 years			
Patients with ≥ 4-point reduction in SGRQ total score, n (%)	121/162 (75)		95/172 (55)
Odds ratio (mepolizumab/placebo), 95% CI		2.80 (1.70, 4.62)	
Lung function: ≤ 60% predicted FEV_1_			
Patients with ≥ 4-point reduction in SGRQ total score, n (%)	171/243 (70)		132/242 (55)
Odds ratio (mepolizumab/placebo), 95% CI		2.13 (1.44, 3.16)	
Lung function: > 60–80% predicted FEV_1_			
Patients with ≥ 4-point reduction in SGRQ total score, n (%)	137/177 (77)		98/176 (56)
Odds ratio (mepolizumab/placebo), 95% CI		3.03 (1.84, 4.99)	
Lung function: ≥ 80% predicted FEV_1_			
Patients with ≥ 4-point reduction in SGRQ total score, n (%)	27/46 (59)		26/47 (55)
Odds ratio (mepolizumab/placebo), 95% CI		2.12 (0.70, 6.48)	

*CI* confidence interval, *FEV*_*1*_ forced expiratory volume in 1 s, *ITT* intent-to-treat, *SGRQ* St George’s Respiratory Questionnaire

**Table 4:** Proportion of patients achieving ≥ 0.5-point reductions in ACQ-5 score, by baseline characteristics

	Mepolizumab		Placebo
ITT population			
Patients with ≥ 0.5-point reduction in ACQ-5 score, n (%)	272/465 (58)		201/462 (44)
Odds ratio (mepolizumab/placebo), 95% CI; p-value		1.91 (1.45, 2.52); p < 0.001	
Age at asthma onset: < 18 years			
Patients with ≥ 0.5-point reduction in ACQ-5 score, n/N (%)	78/132 (59)		50/119 (42)
Odds ratio (mepolizumab/placebo), 95% CI		1.84 (1.06, 3.20)	
Age at asthma onset: 18–< 40 years			
Patients with ≥ 0.5-point reduction in ACQ-5 score, n/N (%)	93/172 (54)		69/171 (40)
Odds ratio (mepolizumab/placebo), 95% CI		1.93 (1.20, 3.12)	
Age at asthma onset: ≥ 40 years			
Patients with ≥ 0.5-point reduction in ACQ-5 score, n/N (%)	100/160 (63)		82/172 (48)
Odds ratio (mepolizumab/placebo), 95% CI		2.27 (1.39, 3.70)	
Lung function: ≤ 60% predicted FEV_1_			
Patients with ≥ 0.5-point reduction in ACQ-5 score, n/N (%)	149/243 (61)		95/238 (40)
Odds ratio (mepolizumab/placebo), 95% CI		2.53 (1.73, 3.71)	
Lung function: > 60–80% predicted FEV_1_			
Patients with ≥ 0.5-point reduction in ACQ-5 score, n/N (%)	101/176 (57)		86/177 (49)
Odds ratio (mepolizumab/placebo), 95% CI		1.40 (0.87, 2.24)	
Lung function: ≥ 80% predicted FEV_1_			
Patients with ≥ 0.5-point reduction in ACQ-5 score, n/N (%)	22/46 (48)		20/47 (43)
Odds ratio (mepolizumab/placebo), 95% CI		1.45 (0.47, 4.48)	

*ACQ* Asthma Control Questionnaire, *CI* confidence interval, *FEV*_*1*_ forced expiratory volume in 1 s, *ITT* intent-to-treat

### Lung function subgroups

Across all lung function subgroups, mepolizumab was associated with reductions of 49–63% in the annual rate of clinically significant exacerbations compared with placebo (Fig. 1A). Furthermore, patients receiving mepolizumab were more likely to experience no clinically significant exacerbations during the study than those who received placebo, irrespective of lung function (Table 2). Compared with placebo, mepolizumab also resulted in an increase from baseline in pre-bronchodilator FEV_1_ plus improvements from baseline in SGRQ total score and ACQ-5 score for all lung function subgroups (Fig. 1B–D). The proportion of patients achieving the MCID from baseline in SGRQ total score was higher with mepolizumab compared with placebo for all lung function subgroups (Table 3). Furthermore, the proportion of patients achieving MCID from baseline in ACQ-5 score was higher with mepolizumab than with placebo for all three subgroups, with a trend towards larger treatment differences with lower % predicted FEV_1_ at baseline (Table 4).

### Airway reversibility subgroups

Mepolizumab was associated with reductions in the annual rate of clinically significant exacerbations compared with placebo in both the reversible and non-reversible airway reversibility subgroups (reductions of 59% [rate ratio 0.41, 95% CI: 0.31, 0.53) and 53% [rate ratio 0.47, 95% CI: 0.32, 0.68], respectively; Fig. 1A).

### Allergen sensitivity subgroups

**Fig. 2:**
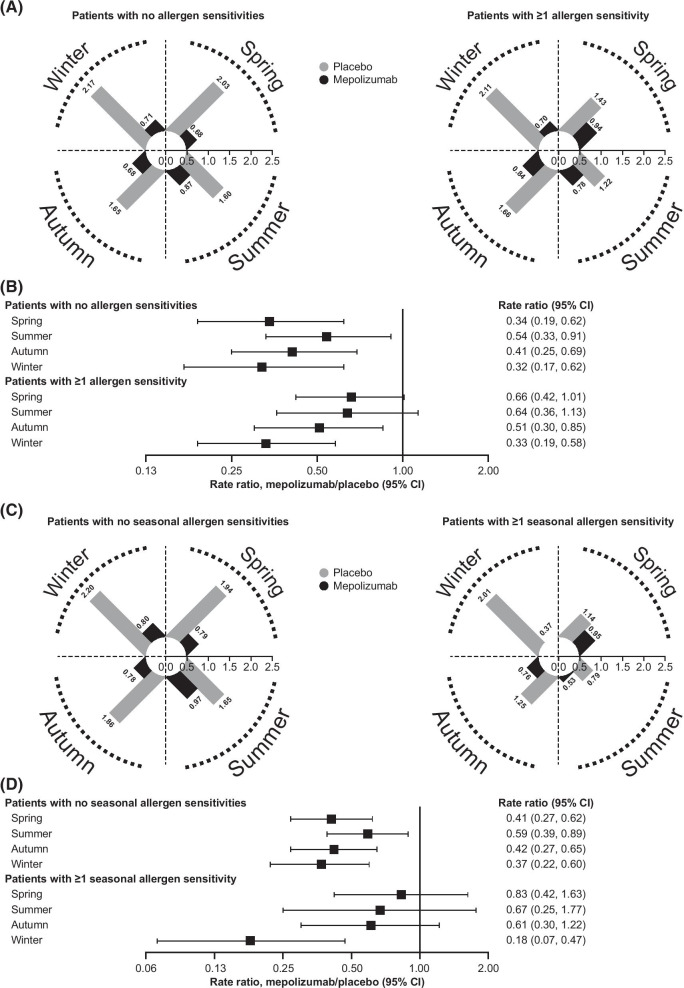
Annualised clinically significant exacerbation rates with mepolizumab versus placebo, by allergen sensitivity. **A** Annualised rate of exacerbations per season, for patients with and without allergen sensitivities receiving mepolizumab and placebo; **B** Rate ratio (mepolizumab versus placebo) of annualised exacerbation rates per season, for patients with and without allergen sensitivities; **C** Annualised rate of exacerbations per season, for patients with and without seasonal allergen sensitivities receiving mepolizumab and placebo; **D** Rate ratio (mepolizumab vs placebo) of annualised exacerbation rates per season, for patients with and without seasonal allergen sensitivities. For all radial plots, each increment of the radial axis represents 0.1 exacerbations/year, with the outer ring representing a possible maximum of 2.5 exacerbations/year. *CI* confidence interval

Throughout the year, patients receiving mepolizumab experienced fewer exacerbations than those receiving placebo, regardless of sensitisation to perennial and seasonal allergens (Fig. 2A–D). Although the annualised rate of exacerbations was relatively consistent across all seasons for patients receiving mepolizumab, the rate fluctuated considerably across seasons for those receiving placebo. In particular, patients with and without allergen sensitivities who received placebo experienced more exacerbations during winter than in other seasons (Fig. 2A, B).

**Fig. 3:**
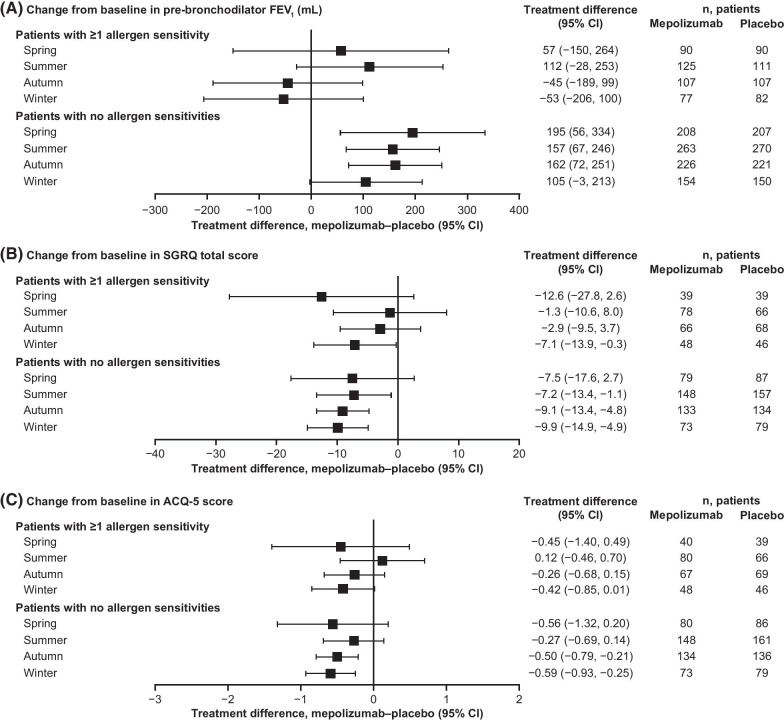
Efficacy of mepolizumab, by allergen sensitivity. **A** Change from baseline to study end in pre-bronchodilator FEV_1_ per season; **B** Change from baseline to study end in SGRQ total score per season; **C** Change from baseline to study end in ACQ-5 score per season. All plots are for patients receiving mepolizumab versus placebo. *ACQ* Asthma Control Questionnaire, *CI* confidence interval, *FEV*_*1*_ forced expiratory volume in 1 s, *SGRQ* St George’s Respiratory Questionnaire

In patients without allergen sensitivities, numerical improvements in pre-bronchodilator FEV_1_ were observed with mepolizumab versus placebo in all seasons (Fig. 3A). In those patients with ≥ 1 allergen sensitivity, mean improvements in pre-bronchodilator FEV_1_ were not seen during the autumn and winter months (Fig. 3A). With regards to HRQoL, although some seasonal fluctuations were seen, mean improvements in SGRQ total score were larger with mepolizumab than with placebo in all seasons, irrespective of allergen sensitivities (Fig. 3B). Similarly, numerical mean improvements in ACQ-5 score were larger with mepolizumab versus placebo in all seasons in patients with no allergen sensitivities, and in all but the summer season in patients with ≥ 1 allergen sensitivity (Fig. 3C).

### Asthma control subgroups

**Fig. 4:**
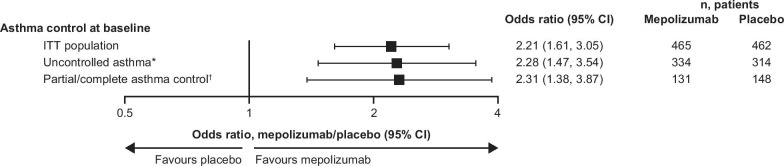
Proportion of patients achieving complete asthma control with mepolizumab versus placebo. Complete asthma control was defined as an ACQ-5 score < 0.75. *Defined as an ACQ-5 score ≥ 1.5; ^†^defined as an ACQ-5 score < 1.5. *ACQ* Asthma Control Questionnaire, *CI* confidence interval

Patients receiving mepolizumab were more likely to achieve complete asthma control at the end of the study compared with those receiving placebo, with similar odds ratios to placebo observed in patients with uncontrolled or partial/complete asthma at baseline (2.28, 95% CI: 1.47, 3.54 and 2.31, 95% CI: 1.38, 3.87, respectively; Fig. 4).

## Discussion

This post hoc analysis of the MENSA and MUSCA trials evaluated the relationship between several baseline clinical characteristics and treatment response to mepolizumab in patients with severe eosinophilic asthma. Consistent benefits were observed with mepolizumab over placebo in several clinically important outcomes, including exacerbations, lung function, HRQoL and asthma control. In general, these improvements were seen across the majority of clinical characteristic subgroups, including those stratified by age of asthma onset, or baseline lung function, airway reversibility, allergen sensitivity, or asthma control.

We found that patients experienced reductions in clinically significant exacerbations plus clinically meaningful improvements in SGRQ total score and ACQ-5 score, irrespective of age at asthma onset or baseline lung function, with seasonal variations in the allergen sensitivity subgroups as discussed further below. Reductions in annualised exacerbation rates were also seen with mepolizumab versus placebo in patients with and without airway reversibility at baseline. While all age of asthma onset subgroups and all lung function subgroups experienced overall numerical improvements in pre-bronchodilator FEV_1_ from baseline, the effect of mepolizumab was highly variable for patients with a FEV_1_ > 80% predicted. Finally, patients with both uncontrolled asthma and partial/complete asthma control at baseline experienced improvements in their asthma control with mepolizumab versus placebo. Overall, our results indicate that subgroups of patients with severe eosinophilic asthma defined by baseline variables of age at asthma onset, lung function, airway reversibility and allergen sensitivities all show similar treatment response to mepolizumab. The results therefore support previous findings that mepolizumab is associated with clinical benefit in a broad population of patients with severe eosinophilic asthma, as detailed below.

The results of this analysis are in line with a previous post hoc analysis of MENSA and MUSCA, which demonstrated that mepolizumab reduced the rate of clinically significant exacerbations and improved SGRQ total and ACQ-5 scores, compared with placebo, in patients with severe eosinophilic asthma across all body weight and body mass index categories [16]. With regards to real-world evidence, results from an interim analysis of the ongoing prospective, observational, global REALITI-A study (which includes patients with severe eosinophilic asthma newly prescribed mepolizumab in routine care) demonstrated that mepolizumab effectively reduces exacerbations in a real-world setting [17]. In addition, reductions in exacerbation rates were observed with mepolizumab among patients with especially severe eosinophilic asthma enrolled in a French early access programme [18]. The patient populations included in these studies represent a wider range of patients with severe asthma than would be included in a randomised clinical trial with stringent inclusion criteria, thus supporting our own observations that mepolizumab is associated with benefit for patients with a wide range of clinical characteristics. Similar to our findings with mepolizumab, treatment with benralizumab has been associated with clinical benefits in patients with subgroups of severe eosinophilic asthma with various clinical characteristics [19]. A number of clinical characteristics were associated with an enhanced response to benralizumab, including % predicted pre-bronchodilator FVC < 65%, age at diagnosis ≥ 18 years and concomitant nasal polyposis [19].

Asthma exacerbations can occur in seasonal cycles for patients of all ages [20–22]. For example, it is expected that for adult patients, asthma exacerbations are often virus-driven during autumn and winter months [20, 22, 23]. Moreover, previous studies indicate that patients with allergen sensitivities are more prone to contracting viral infections and therefore experiencing complications such as severe asthma exacerbations, than those without [24]. Our results suggest that although perennial and seasonal allergies clearly influence the rate of asthma exacerbations throughout the year, mepolizumab appears to have a protective effect on exacerbation rates irrespective of allergies, keeping them stable (and lower than placebo) across all seasons. This is in line with a real-world study conducted by Pelaia et al., which demonstrated that mepolizumab improved exacerbation frequency, OCS use, symptom control and lung function for patients with severe eosinophilic asthma, irrespective of allergic status [25]. Interestingly, we also found that patients receiving placebo experienced more exacerbations during winter than in other seasons, irrespective of their perennial or seasonal allergen sensitivities. Similarly, a post hoc analysis of a Phase IV omalizumab study demonstrated that the rate of asthma exacerbations was greatest during the autumn and spring months in patients treated with placebo, but stable across all seasons in patients treated with omalizumab [26]. It should therefore be noted that in the current study, increased exacerbations seen in the placebo arm during the winter months were likely to be the driver of the larger treatment responses to mepolizumab versus placebo in winter. Indeed, according to recent evidence, biologic therapies that target type 2 inflammation may restore the impaired antiviral response often observed in patients with asthma [23].

In the current study numerical improvements in lung function, HRQoL and asthma control were larger with mepolizumab than with placebo across all seasons in patients without allergen sensitivities. In those patients with allergen sensitivities, treatment benefits with mepolizumab versus placebo fluctuated across the seasons; in particular, mepolizumab was not associated with mean improvements in lung function in these patients during the autumn and winter seasons.

A number of limitations should be considered in the interpretation of these data, in addition to the post hoc nature of the analysis. Owing to the normal distribution of clinical characteristics within a population of patients, there was substantial variation in the number of patients in the different subgroups. Furthermore, the lung function subgroup with pre-bronchodilator FEV_1_ ≥ 80% predicted included a disproportionate number of adolescent patients, owing to the inclusion criteria of the original studies. A small number of events was also observed for some endpoints, such as exacerbations requiring ED visit/hospitalisation; this does not allow for accurate analysis of between-group differences. Moreover, our results are based on a clinical trial population, which may not be fully representative of patients treated in real-world clinical practice. Notably, although patients in the ‘non-reversible’ airway reversibility subgroup for this analysis had a < 12% change in FEV_1_, inclusion criteria for the MENSA and MUSCA studies meant that these patients did have airway hyper-responsiveness on methacholine, histamine or mannitol challenge, or airflow variability [8, 9]; therefore, patients with the most severe asthma who may have fixed airflow obstruction were excluded. Finally, it is important to note that there was no comparison of safety findings across subgroups. Nonetheless, this analysis provides important data on the efficacy of mepolizumab in patients with severe eosinophilic asthma across a broad spectrum of clinical characteristics, which may help to inform clinical decision making.

## Conclusions

The results of this analysis show that the licensed mepolizumab dose (100 mg SC), in addition to optimised standard of care, is associated with improvements in exacerbation rate, lung function, HRQoL, and asthma control in patients with severe eosinophilic asthma irrespective of their age at asthma onset or baseline lung function, airway reversibility, and allergen sensitivities. These data therefore indicate that in patients with severe asthma who have a blood eosinophil count ≥ 150 cells/μL and a history of exacerbations, treatment with mepolizumab is likely to provide clinical benefit across a broad range of clinical characteristics. Although limited to patients eligible for inclusion in randomised controlled trials, our findings are in keeping with those from several real-world studies of mepolizumab in patients with asthma. However, further studies in larger real-world populations than those reported to date will be useful in confirming the results presented here.

## Data Availability

Anonymised individual participant data and study documents can be requested for further research from www.clinicalstudydatarequest.com.

## Abbreviations

ACQ: Asthma Control Questionnaire
CI: Confidence interval
ED: Emergency department
FEV_1_: Forced expiratory volume in 1 s
FVC: Forced vital capacity
HRQoL: Health-related quality of life
ICS: Inhaled corticosteroid
Ig: Immunoglobulin
IL: Interleukin
ITT: Intent-to-treat
IV: Intravenous
MCID: Minimal clinically important difference
OCS: Oral corticosteroid
SC: Subcutaneous
SD: Standard deviation
SGRQ: St George’s Respiratory Questionnaire
